# Symmetrical dimethylarginine is a more sensitive biomarker of renal dysfunction than creatinine

**DOI:** 10.1186/cc12361

**Published:** 2013-03-19

**Authors:** JJ Dixon, K Lane, RN Dalton, IA MacPhee, BJ Philips

**Affiliations:** 1 Acute Kidney Injury Research Group, St George's Hospital and University of London, UK; 2 King's College, University of London, UK

## Introduction

Current definitions of acute kidney injury (AKI) are based upon changes in serum creatinine (SCr) concentration and urine output: both have limitations in patients with AKI. Diagnosis may be delayed if using these criteria alone. We have previously validated a method of measuring the glomerular filtration rate (GFR) intended for use in patients with AKI (a continuous infusion of very low dose iohexol; CIVLDI). In this study we compare the performance of symmetrical dimethylarginine (SDMA) against SCr and CIVILDI. SDMA is the structural isomer of the endogenous nitric oxide inhibitor asymmetrical dimethylarginine. SDMA increases in parallel with SCr. Despite this, no formula exists to estimate GFR from SDMA concentration. Tubular secretion of SCr may be as high as 40%. SDMA may be a more sensitive biomarker of renal dysfunction than SCr; however, the tubular reabsorption of SDMA is unknown. The aims were to compare the performance of SDMA against SCr and accurately measured GFR, and to derive a formula to estimate GFR from SDMA concentration.

## Methods

Seventeen volunteers had GFR measured twice via measuring the clearance of a 5 ml i.v. bolus of iohexol, or CIVLDI. Serum and urine iohexol, Cr and SDMA were measured by high-performance liquid chromatography/tandem mass spectrometry (HPLC-ms/ms) at 10 time points. Fractional excretion of SDMA and SCr were calculated using iohexol as the denominator. SDMA was plotted against measured GFR, and estimated GFR equations were derived from linear, quadratic and third-order polynomial plots.

## Results

Mean GFR measured by single injection was 78.7 ± 28.5 ml/ minute/1.73 m^2^, and 78.9 ± 28.6 ml/minute/1.73 m^2 ^when measured by CIVLDI (*P *= 0.82). Mean SDMA concentration was similar on both occasions (641 ± 38 vs. 623 ± 22 nmol/l; *P *= 0.68). Tubular reabsorption of SDMA was lower in subjects with GFR <60 ml/minute/1.73 m^2 ^(12 ± 8% vs. 18 ± 8% in subjects with GFR >60 ml/minute/1.73 m^2^; *P *= 0.0002), and tubular secretion of SCR was higher in subjects with GFR <60 ml/minute/1.73 m^2 ^(29% vs. 23%; *P *0.0001). The third-order polynomial equation (*r *= 0.93) estimated GFR better than quadratic (*r *= 0.92) and linear (*r *= 0.87) equations. Bland-Altman comparison revealed no bias when the third-order equation was used (precision: ±20 ml/minute/1.73 m^2^).

## Conclusion

SDMA appears to be an accurate and precise estimate of GFR and a more sensitive biomarker of renal dysfunction than SCr. We predict SDMA will perform better than SCr as a biomarker of AKI. This forms the basis of a future study.

